# Cytotoxic activity of standardized extracts, a fraction, and individual secondary metabolites from fenugreek seeds against SKOV-3, HeLa and MOLT-4 cell lines

**DOI:** 10.1080/13880209.2021.1903047

**Published:** 2021-04-13

**Authors:** Justyna Stefanowicz-Hajduk, Barbara Król-Kogus, Barbara Sparzak-Stefanowska, Katarzyna Kimel, J. Renata Ochocka, Mirosława Krauze-Baranowska

**Affiliations:** a Department of Biology and Pharmaceutical Botany, Medical University of Gdańsk, Gdańsk, Poland; b Department of Pharmacognosy with Medicinal Plants Garden, Medical University of Gdańsk, Gdańsk, Poland

**Keywords:** Cancer cells, flavone C-glycosides, RTCA, steroidal saponins, *Trigonella foenum-graecum*

## Abstract

**Context:**

*Trigonella foenum-graecum* L. (Fabaceae) has many therapeutic properties and anticancer potential.

**Objective:**

The cytotoxic activities of standardized extracts and a fraction from fenugreek seeds and their compounds (sapogenins, flavone C-glycosides, alkaloid trigonelline) against human cancer SKOV-3, HeLa and MOLT-4 cells were evaluated.

**Materials and methods:**

Fenugreek seeds were extracted with 70% methanol (A) or water (B). Furthermore, the seeds were purified with petroleum ether and chloroform and next extracted with methanol to obtain fraction (C). The quantitative analysis of saponins and flavonoids in the extracts was done with HPLC methods. The extracts (5–120 µg/mL) and compounds (1–50 µg/mL) were tested on the cells by MTT assay and RTCA system. The effect of a fraction on ROS production, mitochondrial membrane potential and caspase-3/7 activity in HeLa and SKOV-3 cells was also evaluated by flow cytometry.

**Results:**

The strongest cytotoxic activity on cancer cells showed the fraction C (IC_50_ was 3.91 ± 0.03 for HeLa, 3.97 ± 0.07 for SKOV-3, and 7.75 ± 0.37 for MOLT-4) with the highest content of steroidal saponins (163.18 ± 11.03 μg/mg) and flavone C-glycosides (820.18 ± 0.05 μg/mg). The fraction significantly increased ROS production (up to four times higher than in keratinocytes as control) and caspases activity in the cells. The examined flavonoids did not exhibit the cytotoxic activity in contrast to yamogenin, tigogenin, and diosgenin.

**Conclusions:**

The obtained results complement the data on the cytotoxic activity of *Foenugraeci Semen* and synergistic effect of flavonoids and saponins complex contained in the plant.

## Introduction

Fenugreek seeds (*Trigonella foenum-graecum* L., Fabaceae), a popular spice used to modify the taste and colour of food, are also one of the oldest herbal medicines used in many traditional healing systems such as Ayurveda, Traditional Chinese Medicine, and Ancient Egyptian Medicine (Yadav and Baquer 2014; Nagulapalli Venkata et al. 2017; Yao et al. 2020). Nowadays, fenugreek seeds are traditionally applied for lowering blood sugar, and in the treatment of eczema, burns, gout, abdominal distention, diarrhoea and skin inflammation (Bahmani et al. 2016; Yao et al. 2020).

Fenugreek attracts the attention of scientists due to its numerous health benefits, that have been demonstrated and confirmed in animal studies as well as in clinical trials (Bahmani et al. 2016; Nagulapalli Venkata et al. 2017; Yao et al. 2020). Fenugreek seed extracts and their constituents exhibit antidiabetic, antihyperlipidemic, antiobesity, anti-inflammatory, antioxidant, antifungal, and antibacterial properties (Yadav and Baquer 2014; Nagulapalli Venkata et al. 2017; Jabeen et al. 2018; Yao et al. 2020).

Many of the *in vitro* and *in vivo* studies showed the anticancer potential of the fenugreek seeds in experimental models of cancers using cell lines and animals (El Bairi et al. 2017; Nagulapalli Venkata et al. 2017). The numerous studies on fenugreek seeds, which showed their importance in cancer treatment and prevention indicate, that their antitumor activity is due to influence on multiply signalling pathways. Different constituents of *T. foenum-graecum* have been found to target different hallmarks of cancer including proliferation, inflammation, angiogenesis invasion and metastasis, but their mechanism is not always fully understood (Amin et al. 2005).

The pharmacological activity of fenugreek seeds is due to the presence of a significant number of phytoconstituents. So far, over 100 chemical compounds belonging to saponins, flavonoids, and polysaccharides have been isolated from seeds (Yao et al. 2020; Nagulapalli Venkata et al. 2017). It has been proven that among the above-mentioned groups of compounds, the anticancer properties are primarily related to the presence of steroidal sapogenins, mainly diosgenin, and the major component of alkaloid fraction – trigonelline (El Bairi et al. 2017; Chen et al. 2015; Sethi et al. 2018).

Diosgenin, the most abundant aglycone of fenugreek saponins demonstrated significant anticancer potential *in vitro* (Moalic et al. 2001; Corbiere et al. 2004; Melo et al. 2004; Raju et al. 2004; Liu et al. 2005; Raju and Bird 2007; Raju and Mehta 2009; Li et al. 2010; Lepage et al. 2011; Lin et al. 2013; Li et al. 2015; Selim and Al Jaouni 2015). This sapogenin inhibits the proliferation of cancer cells and induced apoptosis in a variety of cancer cell lines including colorectal, osteosarcoma, breast, leukaemia and hepatocellular. Several models of cancer *in vivo* gave evidence that diosgenin inhibits the tumour growth (e.g., rat colorectal tumour, MCF-7 and MDA-MB-231 human breast cancer xenografts in mice, mouse LA795 lung adenocarcinoma tumour) (Chen et al. 2015; El Bairi et al. 2017; Sethi et al. 2018).

Some studies revealed proapoptotic activity of diosgenin glycosides: dioscin (Hsieh et al. 2013; Hu et al. 2013; Lv et al. 2013; Chen et al. 2014; Kim et al. 2014; Zhao et al. 2014; Aumsuwan et al. 2016; Si et al. 2016), protodioscin (Hibasami et al. 2003), methylprotodioscin (Liu et al. 2005; Wang et al. 2006; Bai et al. 2014), tigogenin and yamogenin glycosides (Pettit et al. 1991; Ohtsuki et al. 2004; Lu et al. 2009; Yen et al. 2012), however, very little is known about the cytotoxic activity of other steroidal sapogenins (Corbiere et al. 2003; Trouillas et al. 2005), being aglycones of saponins present in *T. foenum-graecum* seeds. Cytotoxic effect of yamogenin was never evaluated, while for tigogenin only limited data are available (Corbiere et al. 2003; Trouillas et al. 2005).

Trigonelline is a pyridine alkaloid and nicotinic acid betaine derivative. The study revealed that trigonelline suppresses the ROS-induced increase in the invasive capacity of hepatoma cells AH109 without affecting the proliferation of the cells (El Bairi et al. 2017).

This work evaluates for the first time the cytotoxic activity of standardized extracts and fraction from fenugreek seeds against SKOV-3 and MOLT-4 cell lines as well as the activity of selected compounds presenting in them including sapogenins (yamogenin, tigogenin, and diosgenin), flavone C-glycosides and alkaloid trigonelline against cancer SKOV-3 and HeLa cell lines. The influence of the fraction containing flavonoids and saponins on selected cellular factors that play a role in the death of cancer SKOV-3 and HeLa cells was also assessed.

## Materials and methods

### Reagents and standards

Standards of flavone C-glycosides vitexin and orientin were purchased from PhytoLab (Dutendorfer, Germany), vicenin-1, vicenin-2 and vicenin-3 were from WUXI APPTEC (Shanghai, China). Diosgenin and yamogenin were purchased from PhytoLab. Tigogenin was purchased from Sigma-Aldrich (St. Louis, MO, USA). Trigonelline was from Extrasynthese (Lyon, France).

LC-MS-grade acetonitrile and methanol and analytical-grade formic acid were purchased from Merck Millipore (Burlington, MA, USA). Analytical-grade petroleum ether and chloroform were purchased from POCH S.A. (Gliwice, Poland), while trifluoroacetic acid (TFA, 99% purity) from Sigma-Aldrich. Water from Millipore system (Merck Millipore) was used in all experiments.

### Plant material and sample preparation

The seeds of *T. foenum-graecum* of Polish origin were obtained from the herbal company Lewandowski (Kruszynek, Poland, series No F666). Identification of plant material was done by a botanist from the Medicinal Plants Garden of the Medical University of Gdańsk (Poland) (Jolanta Zarembska, MSc) and the voucher specimen (11-009) was deposited in the herbarium of the Medicinal Plants Garden of the Medical University of Gdańsk (Poland).

Dried fenugreek seeds (5.0 g) were ground in an electric grinder and extracted with the use of magnetic stirrer with 70% methanol (2 × 3 h, 2 × 100 mL) at 60 °C (extract A) or boiling water for 5 min (extract B), filtrated and lyophilized (extracts A and B, respectively). Moreover, the fenugreek seeds (10.0 g) were purified by fractionated extraction in a Soxhlet apparatus with the following solvents: petroleum ether and chloroform. Next the plant material was extracted with methanol and concentrated to a volume of 20 mL and placed in the refrigerator (48 h). The resulting precipitate was filtered off and dried in a desiccator (fraction C). Before HPLC analyzes, the extracts A and B, and fraction C – after dissolving it in methanol, were filtered through 0.22 µm nylon syringe filter (ChemLand, Stargard Szczecinski, Poland). In cytotoxicity study, the lyophilized extract A and fraction C were dissolved respectively in dimethyl sulfoxide (DMSO) or methanol at concentration 10 mg/mL and the lyophilized extract B was dissolved in deionized water at concentration 0.375 mg/mL.

Next to the plant extracts, 10 standard compounds present in the seeds were tested, including 6 flavone C-glycosides (vitexin, orientin, isoorientin, vicenin-1, vicenin-2 and vicenin-3), 3 steroid saponins (diosgenin, yamogenin, tigogenin) and an alkaloid (trigonelline). Reference substances were dissolved in methanol at a concentration 1 mg/mL.

### Phytochemical analysis

Chromatographic methods were used for phytochemical studies of steroid saponins and flavonoids in the obtained fenugreek extracts and fractions. Flavonoid analysis was performed by LC-LC-DAD-ESI-MS (2 D LC-DAD-ESI-MS) method (Król-Kogus et al. 2021). Steroidal saponins were analysed and quantified according to an earlier published HPLC-ELSD-ESI-MS method (Król-Kogus et al. 2020).

### Cell line culture

The human cervical (HeLa S3) and ovarian (SKOV-3) adenocarcinomas, acute human lymphoblastic leukaemia (MOLT-4) cell lines and human keratinocytes (HaCaT) were obtained from the American Type Culture Collection (ATCC, Manassas, VA, USA). The HeLa and HaCaT cells were cultured in Dulbecco’s Modified Eagle’s Medium (DMEM), SKOV-3 and MOLT-4 cells were cultured in McCoy’s 5 A and RPMI 1640 Media, respectively. All the media were supplemented with 10% (*v*/*v*) foetal bovine serum (FBS), 2 mM l-glutamine (except for McCoy’s 5 A), 100 units/mL of penicillin, 100 µg/mL of streptomycin (Sigma-Aldrich), and were kept at 37 °C in a humidified 5% CO_2_ incubator.

### MTT assay

The viability of all the cells was determined by 3-(4,5-dimethylthiazol-2-yl)-2,5-diphenyltetrazolium bromide (MTT) assay. The adherent (HeLa, SKOV-3 and HaCaT) as well as suspension (MOLT-4) cells were seeded in 96-well plates at a density of 5 × 10^3^ cells/well and 5 × 10^4^ cells/well, respectively, and treated for 24 h with the extracts (A, B), fraction C, flavonoids, sapogenins and trigonelline at final concentrations of 5.0–120.0 µg/mL, 5.0–100.0 µg/mL and 1.0–50.0 µg/mL, respectively. The range of concentrations was selected experimentally to obtain symmetrical sigmoidal shape of dose-response curves generated by GraFit v. 7 (Erithacus Software, West Sussex, UK). The maximal concentrations of solvents used in the MTT experiments – methanol and DMSO, did not exceed 4.0% (*v/v*) and 0.8% (*v/v*), respectively. Following treatment, the media with the extracts, fraction and compounds were removed and replaced with fresh media with MTT (0.5 mg/mL). After 3 h of incubation, the absorption of the formazan solution was measured at 570 nm with a plate reader (Epoch, BioTek, Winooski, VT, USA). The results are expressed as IC_50_ mean values (±SD, standard deviation) of at least two independent experiments, in six repetitions.

### RTCA cell proliferation assay

The xCELLigence Real-Time Cell Analyzer Dual Plate (RTCA DP, ACEA Biosciences, San Diego, CA, USA) was used for monitoring of viability of adherent cells (HeLa, SKOV-3 and HaCaT) treated with the extracts (A, B), fraction C and sapogenins for 24 h. The cells were seeded at a density of 2 × 10^4^ cells/well into E-plate 16 (ACEA Biosciences). When the cells entered log phase, the extracts, fraction and steroidal saponins were added in the final concentration range of 5.0–120.0 µg/mL, 5.0–100.0 µg/mL, and 1.0–50.0 µg/mL, respectively. The range of concentrations was selected experimentally to obtain symmetrical sigmoidal shape of dose-response curves generated by RTCA software v. 1.2.1 (ACEA Biosciences). The maximal concentrations of the extract A, fraction and compounds solvents in the RTCA experiments were 4.0% (*v/v*) for methanol and 0.8% (*v/v*) for DMSO. To obtain viability profiles and calculate IC_50_ values, we used the RTCA software v. 1.2.1. All the experiments were performed in duplicate, in three independent repeats.

To determine the cytotoxic selectivity of the extracts, fraction and compounds, the selectivity index (SI) was calculated according to the formula:

$$\text{SI}\;={\;\text{IC}}_{50}{}^{\text{(non}-\text{cancer}\;\text{cells)}}/{\text{IC}}_{50}{}^{\text{(cancer}\;\text{cells)}}$$

(Table 1).

**Table 1.** Selectivity indexes of fenugreek extracts and compounds on cancer cell lines.

Extract/Compound		HeLa	SKOV-3	MOLT-4
Fenugreek extract	70% methanol (A)	0.27^a^; 0.52^b^	0.35^a^; 0.65^b^	0.22^a^
	Water (B)	0.96^a^; 1.12^b^	0.99^a^; 1.34^b^	0.63^a^
	Fraction (C)	1.01^a^; 1.21^b^	0.99^a^; 2.14^b^	0.51^a^
Steroid saponins	Yamogenin	0.59^a^; 0.84^b^	0.58^a^; 0.69^b^	n/a
	Tigogenin	0.87^a^; 1.30^b^	<0.62^a^; 1.16^b^	n/a
	Diosgenin	0.76^a^; 0.84^b^	0.64^a^; 1.38^b^	n/a

n/a: not applicable; ^a^SI values obtained from MTT results; ^b^SI values obtained from RTCA results.

SI ≥10 is required for a compound to be considered selective for cancer cells (Table 1) (Quispe et al. 2006; Pena-Moran et al. 2016).

### Evaluation of selected factors in fraction C-induced cell death

The fraction C from *T. foenum-graecum* seeds, which showed the highest cytotoxic activity, was further tested on HeLa and SKOV-3 lines to determine the rate of apoptosis, caspase-3/7 activity, modulation of mitochondrial membrane potential (MMP) and effect on the production of reactive oxygen species (ROS). The cells were incubated in the presence of different concentrations (3, 10, 20, 50, 100 μg/mL) of fraction C for up to 24 h.

### Apoptosis assay

The apoptotic effects of the fraction (fraction C) on HeLa and SKOV-3 cells were estimated using Annexin V and Dead Cell Assay Kit (Merck Millipore). The cells were seeded in 12-well plates at a density of 1 × 10^5^ cells/well and treated with the fraction at concentrations of 3.0–100.0 µg/mL. The fraction solvent – methanol was added to the control cells at a concentration of 1.0% (*v/v*). After treating, the cells were prepared as described previously (Stefanowicz-Hajduk et al. 2016) and analyzed by flow cytometry with Muse Cell Analyzer (Merck Millipore), following the protocol provided by the manufacturer. The experiments were performed in three independent repeats.

### Assessment of mitochondrial membrane potential (MMP)

The Hela and SKOV-3 cells were seeded in 12-well plates at a density of 1 × 10^5^ cells/well and treated with the fraction C at concentrations of 3.0–100.0 µg/mL. The solvent of the fraction (methanol) was added to the control cell sample at a concentration of 1.0% (*v/v*). After 3, 7 and 12 h of incubation, the cells were harvested and prepared using Muse MitoPotential Assay Kit (Merck Millipore) according to the manufacturer’s protocol. The percentage of total depolarized and depolarized/live cells was determined by flow cytometry with Muse Cell Analyzer. All the experiments were independently repeated three times.

### Measurement of reactive oxygen species (ROS) generation

To determine the role of oxidative stress in HeLa and SKOV-3 cells undergoing apoptosis, we treated the cells (1 × 10^5^ cells/well) with the fraction C at concentrations of 3.0–50.0 µg/mL. The concentration of methanol added to the cells (control sample) did not exceed 0.5% (*v/v*). After 24 h, the cells were stained with Muse Oxidative Stress Kit (Merck Millipore), following the manufacturer’s protocol and analyzed by flow cytometry with Muse Cell Analyzer. The results were obtained in three independently repeated experiments.

### Caspase-3/7 activity

To estimate the caspase-3/7 activity, HeLa and SKOV-3 cells were seeded in 12-well plates (1 × 10^5^ cells/well) and treated with the fraction C at concentrations of 3.0–100.0 µg/mL. The control cells were incubated with methanol at a concentration of 1.0% (*v/v*). After 24 h, the cells were harvested and stained using Muse Caspase-3/7 Assay Kit (Merck Millipore) according with the manufacturer’s protocol. The stained cells were analyzed by flow cytometry with Muse Cell Analyzer. The experiments were performed in three independent repeats.

### Statistical analysis

Statistical data were analyzed using the Statistica 12.0 software package (StatSoft. Inc., Tulsa, OK, USA). All data are expressed as mean values ± standard deviation (SD). For comparison studies, Student’s *t-*test was performed. The statistical significance was set at *p* < 0.05.

## Results

### Phytochemical analysis

The content of steroidal saponins in fenugreek seeds extracts (A and B) and fraction C, determined by the HPLC-ELSD-ESI-MS method (Król-Kogus et al. 2020), was: 135.16 ± 4.74 µg/mg of lyophilizate (extract A), 63.13 ± 8.14 µg/mg of lyophilizate (extract B) and 163.18 ± 11.03 µg/mg of lyophilizate (fraction C).

The content of flavone C-glycosides in fenugreek seeds extracts (A and B) and fraction C determined by 2 D LC-DAD-ESI-MS was 113.37 ± 0.12 µg/mg of lyophilizate (extract A), 36.77 ± 0.29 µg/mg (extract B) and 820.18 ± 0.05 µg/mg (fraction C), respectively (Król-Kogus et al. 2021).

### Cytotoxic activity

Cytotoxic activity of different extracts (A, B) and fraction C from fenugreek seeds and the selected biologically active secondary metabolites (10 standards) was evaluated against three cancer cell lines – SKOV-3, HeLa and MOLT-4.

According to the National Cancer Institute (NCI) guidelines, compounds/extracts/fractions can be considered as cytotoxic if IC_50_ values are under 20–30 μg/mL (Boik 2001). Based on the MTT results, the extracts and fraction, as well as yamogenin and diosgenin were recognized as cytotoxic to the tested cell lines. On the other hand, all analyzed flavone C-glycosides (vitexin, orientin, isoorientin, vicenin-1, vicenin-2 and vicenin-3) and trigonelline alkaloid were found to be inactive (IC_50_ >50 μg/mL) (Table 2).

**Table 2.** IC_50_ values of the fenugreek extracts, flavonoid fraction and pure compounds’ cytotoxic activity on HaCaT, HeLa, SKOV-3 and MOLT-4 cell lines based on the MTT assay.

Sample type	Extracts, flavonoid fraction and pure compounds	IC_50_ (μg/mL) ± SD			
		HaCaT	HeLa	SKOV-3	MOLT-4
Fenugreek extract	70% methanol (A)	3.58 ± 0.21	13.47 ± 0.62	10.34 ± 0.16	16.18 ± 1.14
	Water (B)	16.67 ± 0.22	17.43 ± 0.3	16.68 ± 0.6	26.55 ± 0.07
	Fraction (C)	3.94 ± 0.04	3.91 ± 0.03	3.97 ± 0.07	7.75 ± 0.37
Steroid saponins	Yamogenin	9.70 ± 0.91	16.5 ± 0.59	16.70 ± 0.08	n.t.
	Tigogenin	31.10 ± 2.31	35.60 ± 3.69	>50	n.t.
	Diosgenin	12.40 ± 0.5	16.30 ± 0.26	19.30 ± 0.97	n.t.
Flavone C-glycosides	Vitexin	>50	n.t.	>50	n.t.
	Orientin	>50	>50	>50	n.t.
	Isoorientin	>50	>50	>50	n.t.
	Vicenin-1	>50	>50	>50	n.t.
	Vicenin-2	>50	>50	>50	n.t.
	Vicenin-3	>50	>50	>50	n.t.
Alkaloid	Trigonelline	>50	>50	>50	n.t.

**Figure 1.**
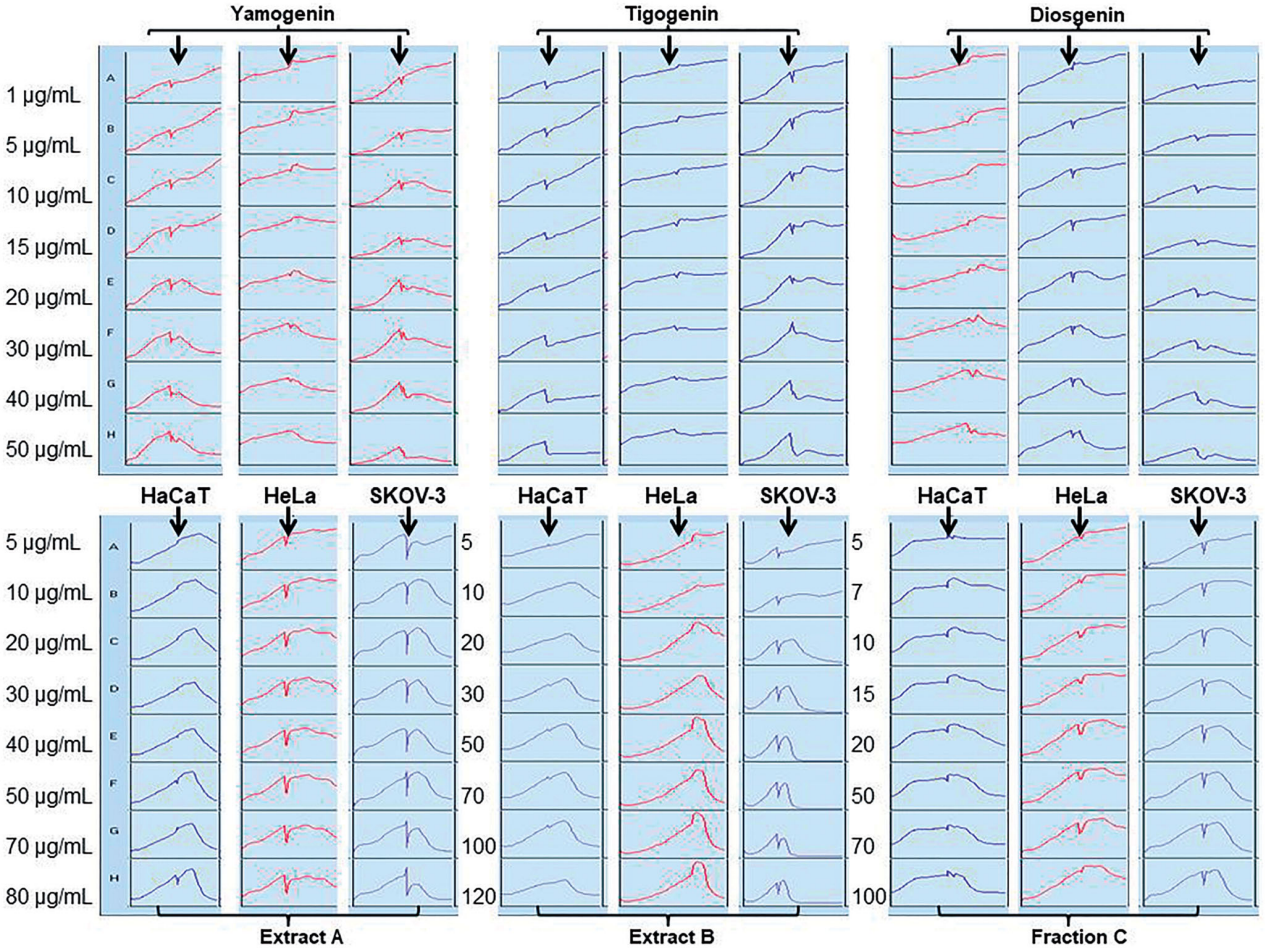
The viability profiles of HaCaT, HeLa and SKOV-3 cells treated with the extracts or compounds of *T. foenum-graecum* obtained by RTCA system. The cells were incubated with sapogenins, methanol (extract A), water extract (extract B) or fraction C at concentrations of 1–50 and 5–120 μg/mL for 24 h, respectively. The black arrows indicate when the compounds or extracts were added to the cells.

To confirm the MTT results for the extracts and compounds with the significant activity (IC_50_ values <50 µg/mL), we performed experiments with RTCA system (Figure 1 and Table 3), which is based on impedance changes in microsensors and allows real-time and continuously monitoring of cell proliferation. This system enables observation of cellular changes at each measuring point of an experiment (Kustermann et al. 2013).

**Table 3.** IC_50_ values of the fenugreek extracts, flavonoid fraction and pure compounds’ cytotoxic activity on HaCaT, HeLa and SKOV-3 cell lines based on the xCELLigence system (RTCA).

Sample type	Extracts, fraction and pure compounds	IC_50_ (μg/mL) ± SD					
		HaCaT	*R* ^2^	HeLa	*R* ^2^	SKOV-3	*R* ^2^
Fenugreek extract	70% methanol (A)	5.06 ± 0.36	0.54	9.65 ± 0.78	0.83	7.73 ± 0.1	0.98
	Water (B)	35.26 ± 0.34	0.99	31.45 ± 0.21	0.97	26.40 ± 0.71	0.98
	Fraction (C)	4.84 ± 1.22	0.89	3.99 ± 0.26	0.97	2.26 ± 0.78	0.90
Steroid saponins	Yamogenin	16.40 ± 1.41	0.94	19.60 ± 1.41	0.92	23.90 ± 1.48	0.97
	Tigogenin	32.70 ± 0.07	0.89	25.10 ± 0.12	0.85	28.30 ± 3.46	0.95
	Diosgenin	23.40 ± 0.42	0.91	28.00 ± 2.40	0.87	16.90 ± 2.89	0.91

*R*^2^: coefficient of determination.

The obtained data were in majority consistent between both experiments, however some differences in IC_50_ values observed in both methods may be due to the fact that MTT results were obtained in one selected time point, while in RTCA system IC_50_ values were calculated from entire time range of treating the cells with the extracts or sapogenins. Furthermore, the RTCA system is very sensitive to any changes in impedance values during the whole experiment.

### Steroid sapogenins effects on proliferation and viability of the tested cell lines

Among the steroidal sapogenins analyzed, based on the MTT results (Table 2), the most cytotoxic against HeLa cells were yamogenin (IC_50_ = 16.5 ± 0.59 μg/mL) and diosgenin (IC_50_ = 16.3 ± 0.26 μg/mL), whereas tigogenin showed almost two times lower activity (IC_50_ = 35.6 ± 3.69 μg/mL). The results obtained using the RCTA system indicates that the cytotoxic activity of sapogenins on HeLa line was in the range of 20–30 µg/mL (Table 3).

Inhibition of SKOV-3 cells proliferation by diosgenin and yamogenin was similar in the MTT test with IC_50_ values for each compound: 19.3 ± 0.97 μg/mL and 16.7 ± 0.08 μg/mL, respectively. These activities in RTCA experiments were also about 20 µg/mL. Tigogenin had the lowest activity in both methods (Tables 2 and 3).

### Cytotoxic effects of fraction C of methanol extract on the tested cell lines

The results of cytotoxic activity of extracts and fraction from *T. foenum-graecum* seeds assessed on HeLa, SKOV-3 and MOLT-4 cell lines indicated that among the analyzed samples, the fraction C from methanol extract (fraction C) showed the strongest effect on the cells with IC_50_ values: 3.91 ± 0.03 μg/mL (MTT) and 3.99 ± 0.26 μg/mL (RCTA) on HeLa cells, 3.97 ± 0.07 μg/mL (MTT) and 2.26 ± 0.78 μg/mL (RCTA) on SKOV-3 cells, 7.75 ± 0.37 μg/mL (MTT) on MOLT-4 cells. The 70% methanol (A) and water (B) extracts showed significantly lower activity on all the cell lines (Tables 2 and 3).

To estimate the selectivity of extracts, fraction and compounds with cytotoxic activity (and IC_50_ <50 μg/mL) we calculated the selectivity index (SI) according to the formula described in the Materials and methods section. All the tested extracts and compounds have SI values below 10, thus according to the selectivity criteria and obtained SI values none of the extracts/fraction/compounds is selective (Quispe et al. 2006; Pena-Moran et al. 2016) (Table 1).

### Apoptotic effects of fraction C on the tested cell lines

**Figure 2.**
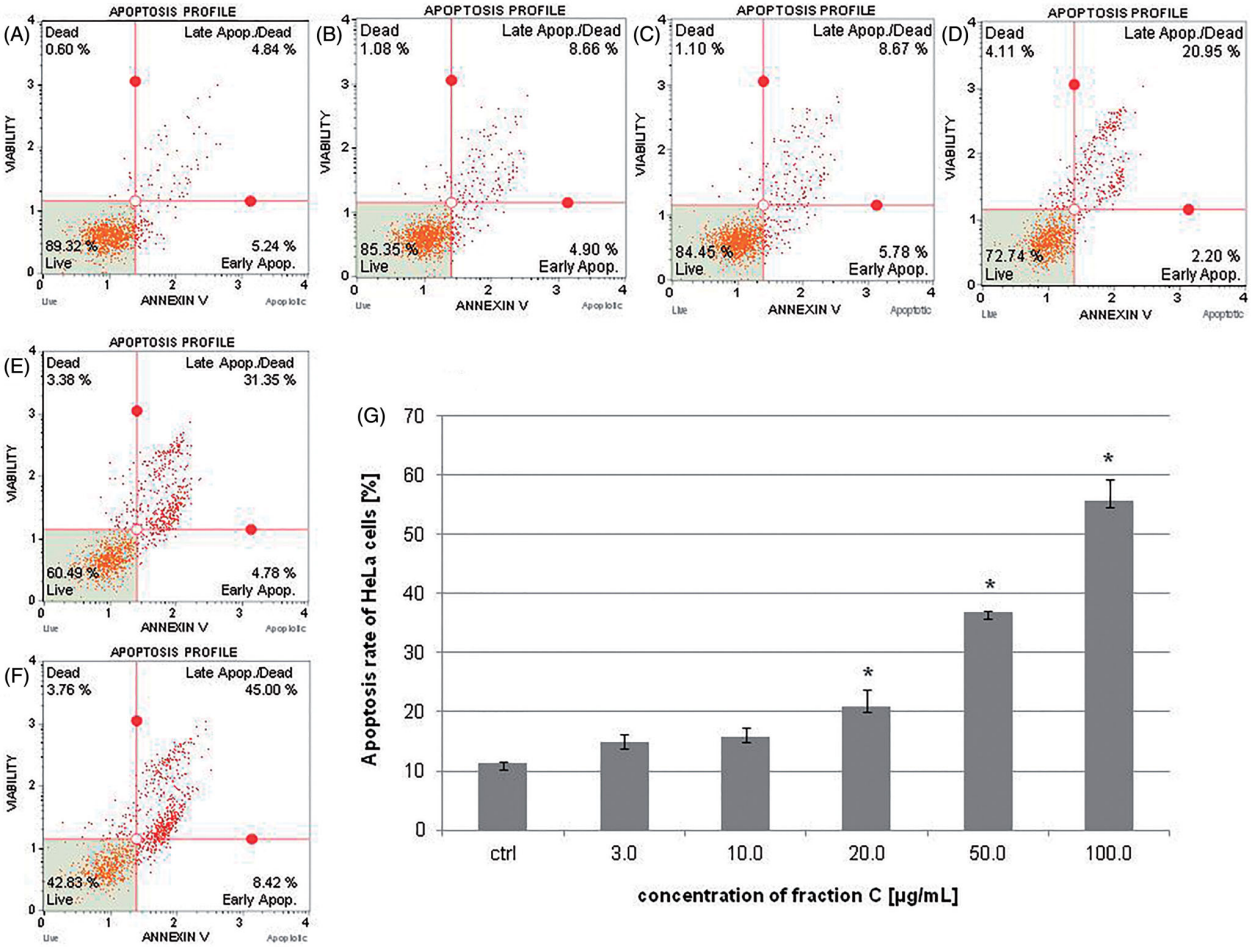
The percentage of early and late apoptotic HeLa cells (apoptosis rate) determined by flow cytometry. The cells were treated with methanol (1% *v/v*) as control (ctrl, A) and fraction C at the concentrations of 3 (B), 10 (C), 20 (D), 50 (E), and 100 µg/mL (F) for 24 h. The results are presented as mean values of three independent repeats (G). Error bars represent standard deviations. Significant differences relative to the control are marked with an asterisk ‘*’ (*p* < 0.05).

**Figure 3.**
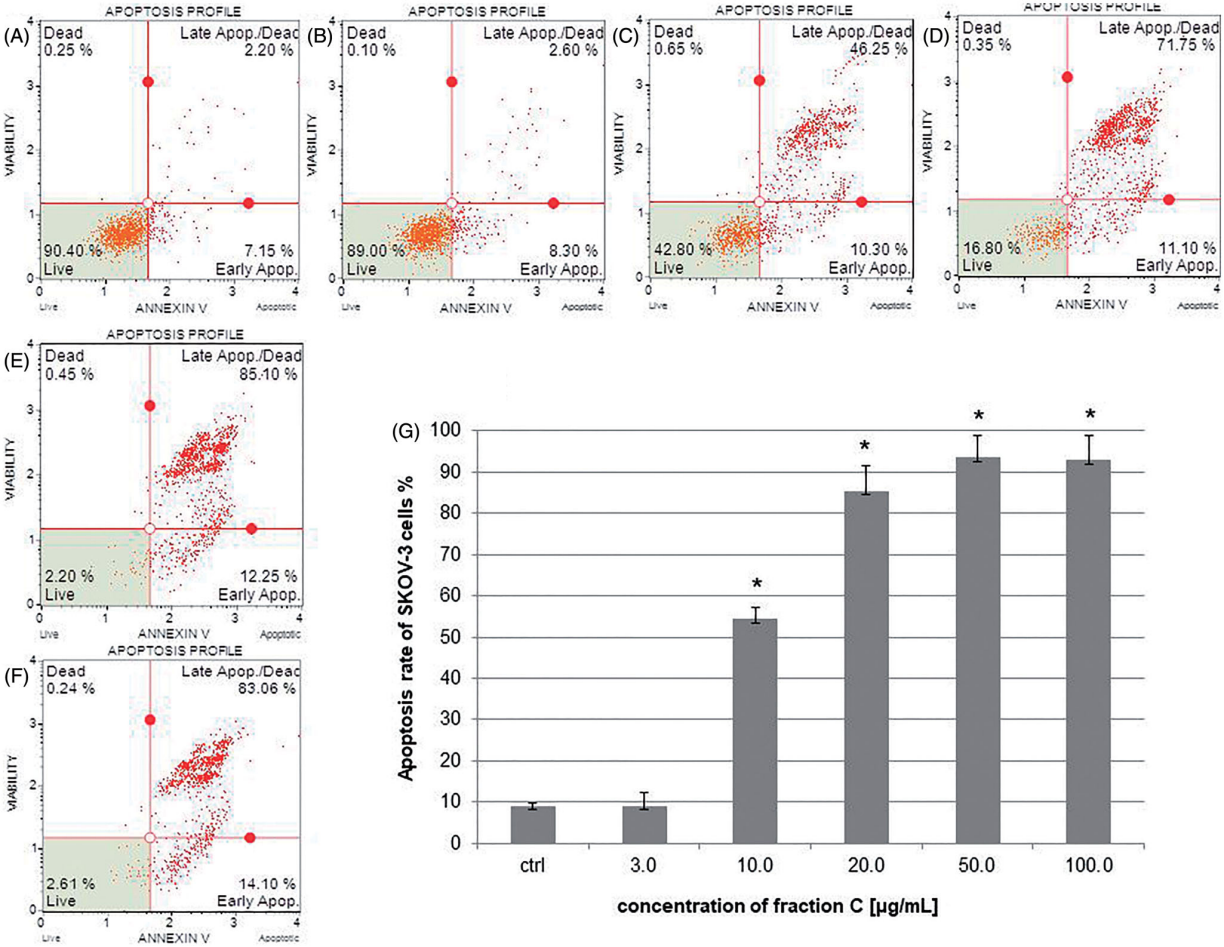
The percentage of early and late apoptotic SKOV-3 cells (apoptosis rate) determined by flow cytometry. The cells were treated with methanol (1% *v/v*) as control (ctrl, A) and fraction C at the concentrations of 3 (B), 10 (C), 20 (D), 50 (E), and 100 µg/mL (F) for 24 h. The results are presented as mean values of three independent repeats (G). Error bars represent standard deviations. Significant differences relative to the control are marked with an asterisk ‘*’ (*p* < 0.05).

The apoptotic effect was estimated after 24 h of treatment the cells with fraction C from *T. foenum-graecum* seed extract. The obtained results indicated that this effect increased in a dose-dependent manner and was stronger in SKOV-3 than HeLa cell line. The amount of early and late apoptotic cells (apoptosis rate) for HeLa cells was 14.79 ± 1.41, 15.85 ± 1.48, 20.94 ± 2.74, 36.82 ± 0.17, and 55.63 ± 3.58% for the fraction concentrations of 3.0, 10.0, 20.0, 50.0, and 100.0 μg/mL, respectively (Figure 2). In the case of SKOV-3 cells, the percentage of early and late apoptotic cells was: 9.06 ± 3.36, 54.35 ± 2.83, 85.44 ± 5.93, 93.55 ± 5.38, and 92.91 ± 6.01% for the fraction concentrations of 3.0, 10.0, 20.0, 50.0, and 100.0 μg/mL, respectively (Figure 3).

### Fraction C effects on the activity of caspase-3/7 in HeLa and SKOV-3 cell lines

**Figure 4.**
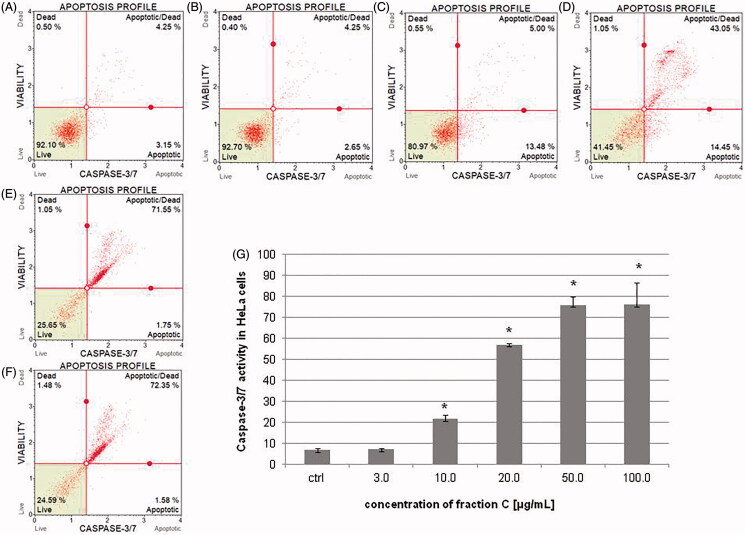
The activity of caspase-3/7 measured after 24 h of incubating the HeLa cells with fraction C. The cells were treated with methanol (1% *v/v*) as control (ctrl, A) and fraction C at the concentrations of 3 (B), 10 (C), 20 (D), 50 (E), and 100 µg/mL (F). The results are presented as mean values of three independent repeats (G). Error bars represent standard deviations. Significant differences relative to the control are marked with an asterisk ‘*’ (*p* < 0.05).

**Figure 5.**
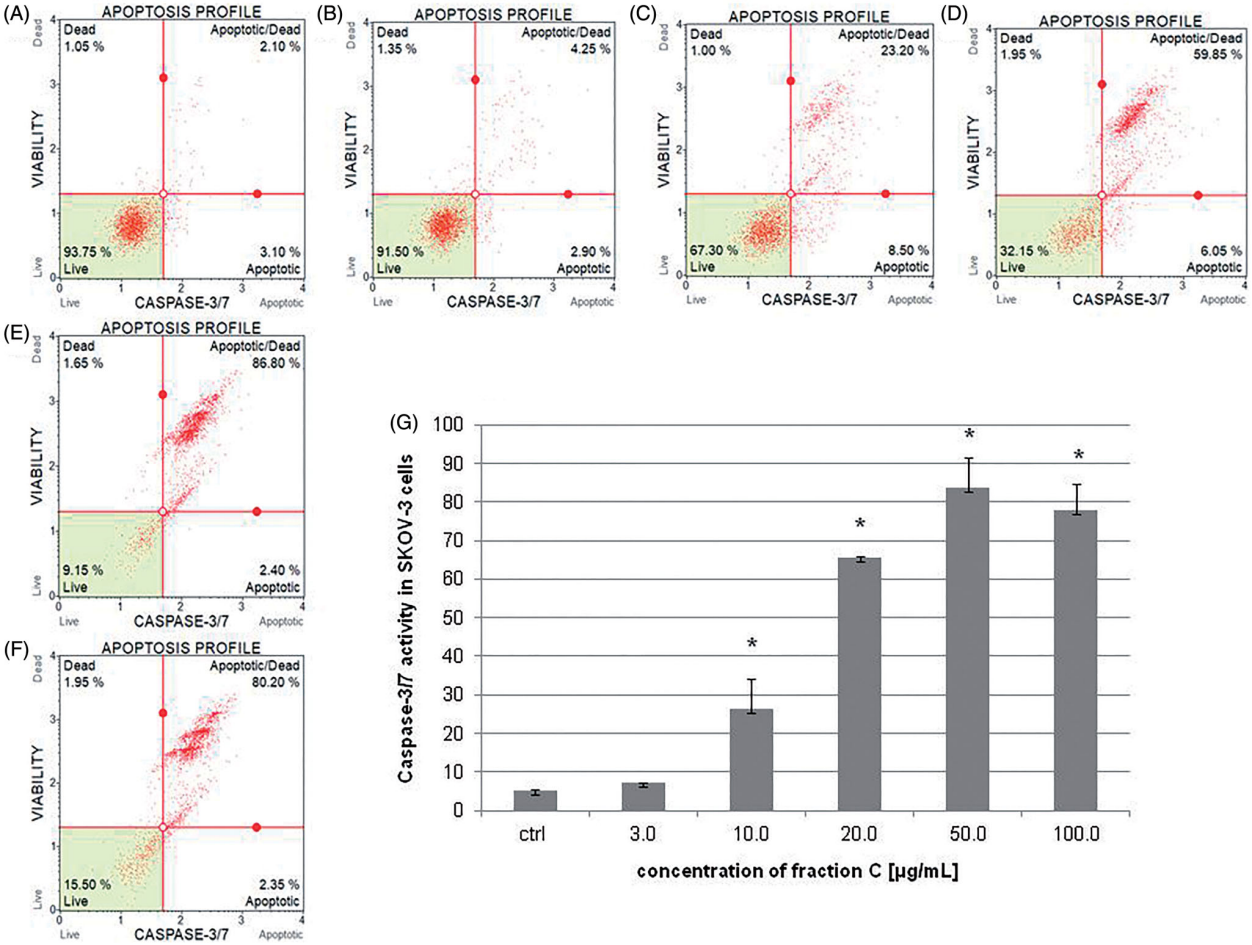
The activity of caspase-3/7 measured after 24 h of incubating the SKOV-3 cells with fraction C. The cells were treated with methanol (1% *v/v*) as control (ctrl, A) and fraction C at the concentrations of 3 (B), 10 (C), 20 (D), 50 (E), and 100 µg/mL (F). The results are presented as mean values of three independent repeats (G). Error bars represent standard deviations. Significant differences relative to the control are marked with an asterisk ‘*’ (*p* < 0.05).

To determine the percentage of cells at various stages of apoptosis based on executioner caspase activity, the cells were treated with different concentrations of fraction C for 24 h. The results obtained with flow cytometry technique showed that fraction C increased caspase-3/7 activity in a dose-dependent manner in both cell lines. The activity of the caspases is presented as percentage of apoptotic cells in the treated cells populations. The results for HeLa cells were 7.25 ± 0.49, 21.32 ± 2.87, 57.0 ± 0.71, 75.95 ± 3.75, and 76.18 ± 10.29% for the fraction concentrations of 3.0, 10.0, 20.0, 50.0, and 100.0 μg/mL, respectively (Figure 4). In the case of SKOV-3 cells, the percentage of apoptotic cells was: 7.08 ± 0.11, 26.15 ± 7.85, 65.70 ± 0.28, 83.68 ± 7.81, and 77.93 ± 6.61% for the fraction concentrations of 3.0, 10.0, 20.0, 50.0, and 100.0 μg/mL, respectively (Figure 5). The involvement of executioner caspases in the observed death process of the cancer cells exposed to the fraction C confirms the induction of apoptosis in HeLa and SKOV-3 cell lines.

### Fraction C effects on mitochondrial membrane potential (MMP) in HeLa and SKOV-3 cell lines

**Figure 6.**
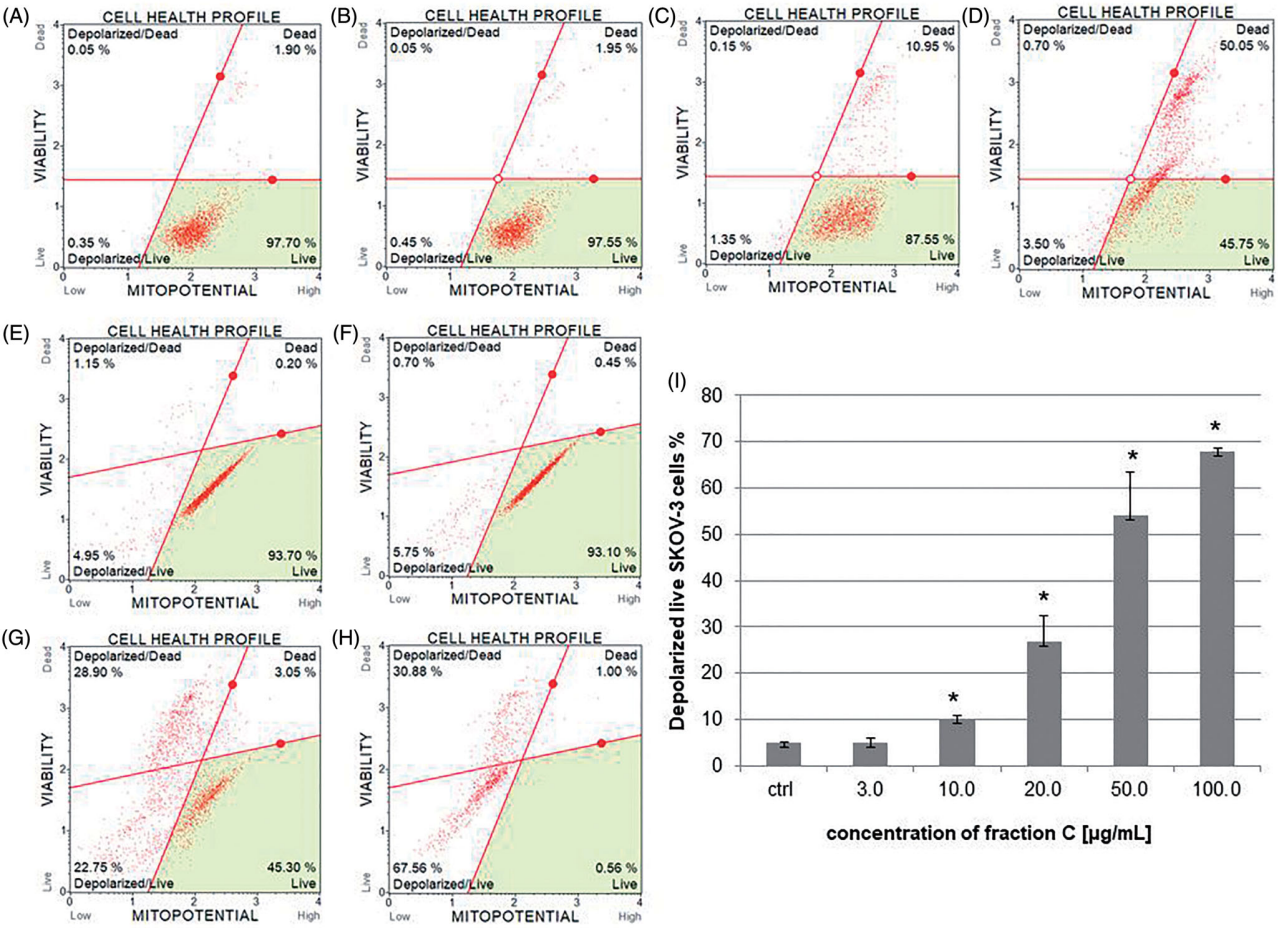
The percentage of depolarized live HeLa and SKOV-3 cells after treating with fraction C. The HeLa (A–D) and SKOV-3 (E–H) cells were incubated for 12 h with methanol (1% (*v/v*)) as control (ctrl, A, E) and fraction C at the concentrations of 3 (B, F), 20 (C, G), and 100 µg/mL (D, H). The amount of depolarized SKOV-3 cells are presented as mean values of three independent repeats (I). Error bars represent standard deviations. Significant differences relative to the control are marked with an asterisk ‘*’ (*p* < 0.05).

HeLa and SKOV-3 cells were treated with different concentrations of fraction C for 3-12 h and the state of mitochondrial dysfunction of the cells was determined. The loss of mitochondrial inner transmembrane potential is often observed in the early stages of apoptosis (Ly et al. 2003). SKOV-3 cells incubated with increasing concentrations of fraction C (3–100 μg/mL) showed a dose-dependent decrease in fluorescence compared to high fluorescence of control cells (exposed to 1% methanol). The percentage of depolarized live SKOV-3 cells was: 4.98 ± 1.10, 10.12 ± 0.74, 26.69 ± 5.57, 53.98 ± 9.45, and 67.97 ± 0.57% for the fraction concentrations of 3.0, 10.0, 20.0, 50.0, and 100.0 μg/mL, respectively. No significant changes in MMP were observed in HeLa cells after 12 h of treating the cells with the fraction as well as after 3 and 7 h (Figure 6).

### Fraction C effects on the production of reactive oxygen species (ROS) in HeLa and SKOV-3 cell lines

**Figure 7.**
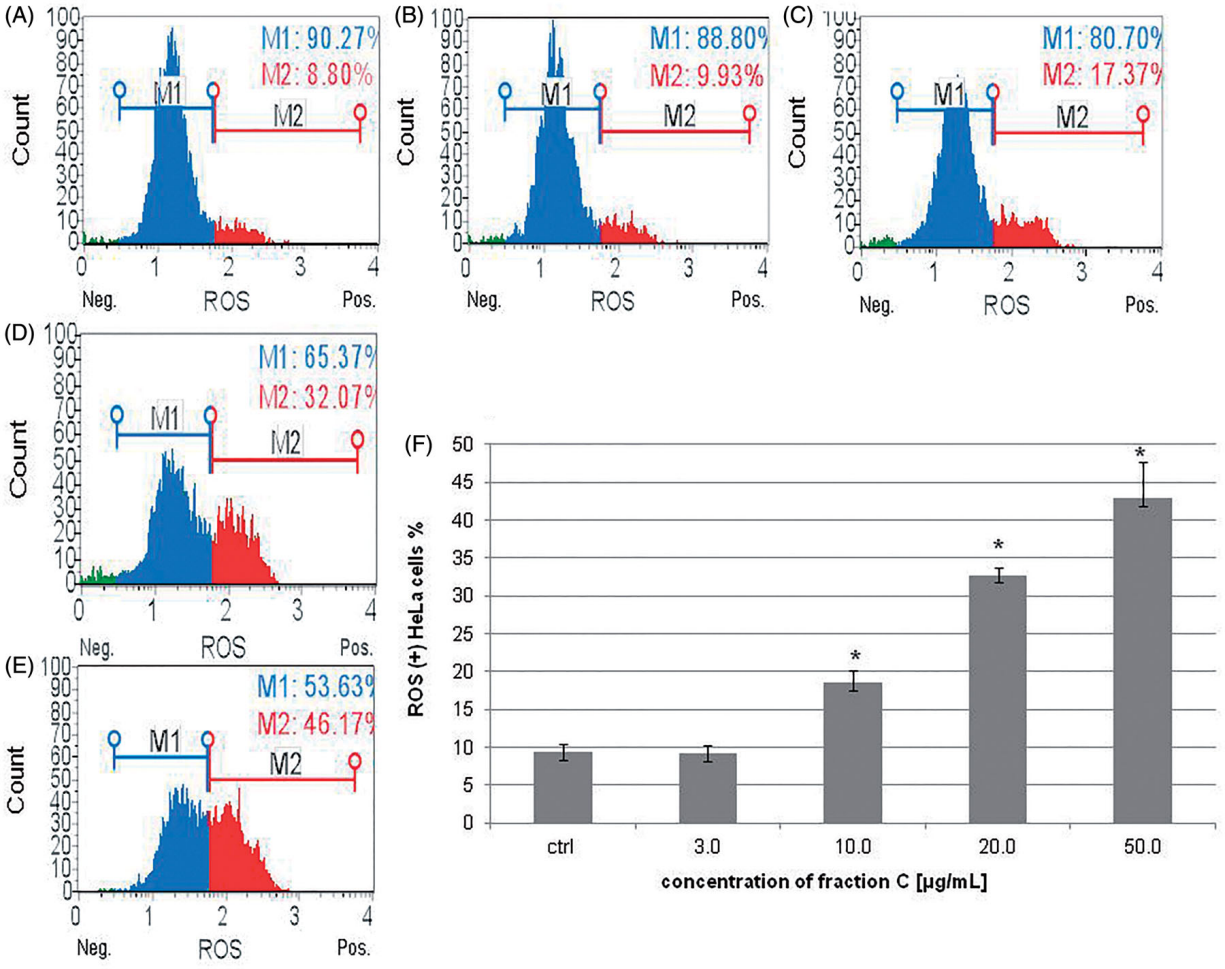
The changes observed in ROS production in HeLa cells incubated with fraction C for 24 h. The cells were treated with methanol (1% *v/v*) as control (ctrl, A) and fraction C at the concentrations of 3 (B), 10 (C), 20 (D), and 50 µg/mL (E). The results are presented as mean values of three independent repeats (F). Error bars represent standard deviations. Significant differences relative to the control are marked with an asterisk ‘*’ (*p* < 0.05).

**Figure 8.**
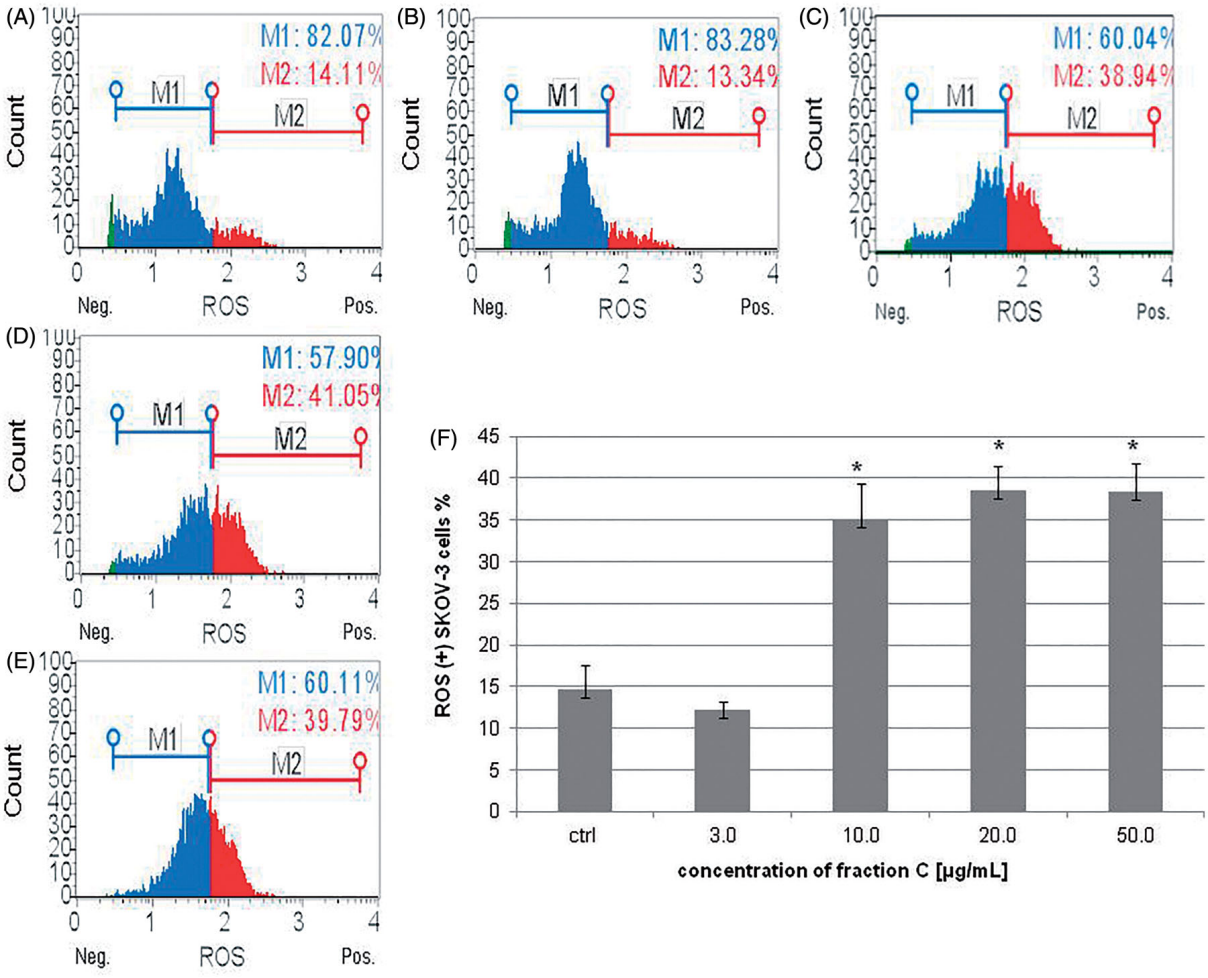
The changes observed in ROS production in SKOV-3 cells incubated with fraction C for 24 h. The cells were treated with methanol (1% *v/v*) as control (ctrl, A) and fraction C at the concentrations of 3 (B), 10 (C), 20 (D), and 50 µg/mL (E). The results are presented as mean values of three independent repeats (F). Error bars represent standard deviations. Significant differences relative to the control are marked with an asterisk ‘*’ (*p* < 0.05).

ROS production was measured in HeLa and SKOV-3 cells after 24 h of treatment with fraction C at various concentrations. Determination of the number and percentage of cells exposed to oxidative stress (ROS(+) cells) was based on the intracellular detection of superoxide radicals. The obtained results indicated a significant increase in ROS production in both cell lines, confirming the role of oxidative stress in the cells death. The percentage of ROS positive (+) cells in the treated HeLa population was: 9.15 ± 1.1, 18.5 ± 1.6, 32.74 ± 0.94, 42.84 ± 4.75% for the fraction concentrations of 3.0, 10.0, 20.0, and 50.0 μg/mL, respectively (Figure 7). In the case of SKOV-3 population, the percentage of ROS positive (+) cells was: 12.28 ± 0.93, 35.11 ± 4.24, 38.57 ± 2.93, 38.36 ± 3.41% for the fraction concentrations of 3.0, 10.0, 20.0, and 50.0 μg/mL, respectively (Figure 8).

## Discussion

As a result of the experiments carried out using the MTT test and RTCA system, the cytotoxic effects of the following methanol extract fraction (C), 70% methanol extract (A) and water extract (B) from fenugreek seeds of Polish origin and selected steroid sapogenins against three human cancer cell lines were revealed. The determined active concentrations generally meet the guidelines of the American NCI regarding plant extracts classified as cytotoxically active (IC_50_ < 20-30 µg/mL) (Boik 2001). In our study, the cytotoxic activity of *T. foenum-graecum* against MOLT-4 and SKOV-3 cell lines was evaluated for the first time. The obtained IC_50_ values​ against the HeLa cell line were significantly lower than the IC_50_ value determined by Aktas and Altun (2015) for methanol extract from *T. foenum -graecum* seeds (IC_50_ = 84.0 ± 0.34 μg/mL).

Tested steroidal sapogenins, 70% methanol extract (A) and aqueous extract (B), and the fraction C from *Foenugraeci Semen* did not show selective effect on the HeLa, SKOV-3 and MOLT-4 tumour cell lines compared to the control HaCaT keratinocytes line. In studies by Stefanowicz-Hajduk et al. (2015), saponins from *Paris quadrifolia* L. (Liliaceae) also showed cytotoxic activity against the human keratinocyte HaCaT control line. Similar results were obtained by Shabbeer et al. (2009) showing the cytotoxic activity of diosgenin on both prostate tumour cells (DU-145, LNCaP, PC-3) and the control line of normal prostate epithelial cells. However, *T. foenum-graecum* seed extract in these studies revealed a selective cytotoxic activity on the above-mentioned three prostate cancer cell lines. Moreover, Al-Oqail et al. (2013), observed a toxic effect of fenugreek seed ether extract on control cell lines in addition to the cytotoxic effect on Hep-2 and MCF-7 cell lines. On the other hand, methanol and aqueous extracts of fenugreek seeds revealed selective cytotoxic activity, respectively on T-cell, B-cell lymphoma, papillary thyroid (FRO), breast cancer cell lines (Alsemari et al. 2014) and HeLa, Mat-LyLu rat prostate cancer lines (Aktas and Altun 2015), without affecting control lines (human lymphocyte and 3T3 mouse fibroblast lines, respectively).

Among the compounds tested against the above-mentioned cell lines (flavone C-glycosides, sapogenins, trigonelline), only saponins were active, including diosgenin, tigogenin, and yamogenin.

So far, many studies have focussed on the cytotoxic activity of diosgenin (Moalic et al. 2001; Corbiere et al. 2004; Melo et al. 2004; Raju et al. 2004; Liu et al. 2005; Raju and Bird 2007; Raju and Mehta 2009; Li et al. 2010; Lepage et al. 2011; Lin et al. 2013; Li et al. 2015; Selim and Al Jaouni 2015), however, the effect of this sapogenin and tigogenin, and yamogenin on the SKOV-3 cell line was not assessed previously.

The IC_50_ value of diosgenin for HeLa cell line obtained in our study in the MTT assay is consistent with other previous studies (Wang et al. 2002; Huo et al. 2004). Cytotoxic activity has also been revealed for glycoside derivative of diosgenin – dioscin (El Bairi et al. 2017). Antiproliferative effect of tigogenin against the osteomatous 1547 cell line has only been found (Corbiere et al. 2003; Trouillas et al. 2005). The cytotoxic activity of glycosidic forms of tigogenin from *Agave fourcroydes* Lem. (Asparagaceae) was described. However, Ohtsuki et al. (2004), in the study of the steroid saponin complex, noted that sapogenin hexa-glycosides show proapoptotic activity against the HeLa line, while the di-glycoside forms remain inactive.

The cytotoxic properties of free yamogenin have not been studied as well, although research has been conducted on its glycosidic forms, e.g., the activity of yamogenin 3-*O*-β-d-glucosyl(6→1)-3-*O*-β-D-glucoside, isolated from *Solanum torvum* Sw. (Solanaceae) was evaluated against a number of cell lines, including gastric cancer MCG-803, hepatocellular Hep2G2, lung cancer A549 and breast cancer MCF-7 (Pettit et al. 1991; Lu et al. 2009; Yen et al. 2012). Our studies have shown, that yamogenin exhibits significant cytotoxicity against HeLa and SKOV-3 cell lines. The obtained results are the first data on the cytotoxic activity of yamogenin.

Yamogenin, similarly to diosgenin, possess in the structure 5,6-double bond. It is speculated, that this bond probably played a significant role in biological effect of saponins. However, absence of the 5,6-double bond could be compensated by other structural characteristics like 5β conformation (Podolak et al. 2010). In the study of the role of 5,6-double bond in cytotoxic effect on human 1547 osteosarcoma cell (Trouillas et al. 2005) it was revealed that sapogenins without 5,6-double bond, namely tigogenin (stereoisomer 5α) and its stereoisomer (5β) – smilagenin only weakly induced apoptosis, compared to diosgenin. Both diosgenin and smilagenin strongly inhibited cell proliferation and arrested cell cycle in G0/G1 phase, whereas tigogenin did not induce this effect (Trouillas et al. 2005). Our obtained results confirmed that the presence of the 5,6-double bond might be partially responsible for the biological activity of particular saponins. This seems to be confirmed by the fact that tigogenin possessed the lowest cytotoxic activity against all analyzed cell lines.

The differences in the content of steroidal saponins between the 70% methanol (A) and aqueous (B) extracts are small and seem not directly related to differences in their cytotoxic activities but it should be emphasized that in our study the cytotoxic activity of free sapogenins was only assessed, whereas in the analyzed fenugreek seed extracts their glycosidic forms are present, for which antitumor and proapoptotic activity has been proved (Hibasami et al. 2003; Wang et al. 2004; Liu et al. 2005; Wang et al. 2006; Podolak et al. 2010; Tong et al. 2012; Waheed et al. 2012; Bai et al. 2014). Therefore, the observed cytotoxic activity of the tested extracts and fraction does not have to be correlated only with the activity of free sapogenins. In fraction C, 70% of the saponin complex was constituted by glycoside forms of diosgenin, and 21% by glycoside forms of yamogenin (calculated on the basis of peak areas on obtained HPLC chromatograms) (Król-Kogus et al. 2020). Their cytotoxic activity was not the subject of this study.

Interestingly, in the MTT test, the cytotoxic activity of diosgenin is very similar to that of the aqueous extract (B), which is probably related to the presence of other secondary metabolites, mainly flavone C-glycosides and the resulting synergism of action. The synergistic effect is probably also responsible for the fact that the cytotoxic effect of fraction C is much stronger than that of the single most active substance.

The flavone C-glycosides exhibit a wide range of benefits for human health, which are the result of their significant antioxidant, anticancer, hepatoprotective and anti-inflammatory activities. They were found to be more stable than aglycones or flavonoid *O*-glycosides. The flavone C-glycosides are poorly absorbed in humans with very few metabolites in urine and blood, however the intestinal bacteria can cleave the C-glycosyl bond in different flavone C-monoglycosides such as orientin, vitexin or isovitexin, which are deglycosylated to their aglycones and degraded (Xiao et al. 2016).

Next to the antioxidant and free radical scavenging activity of flavonoids, there are several studies, which have revealed that anticancer properties of flavonoids might be connected with prooxidant activity, which can cause oxidative damage by reacting with various biomolecules, such as lipids, proteins and DNA. Cancer cells exhibit a higher and more persistent oxidative stress level compared to normal cells, rendering malignant cells more vulnerable to being killed by drugs increasing cellular ROS level, such as some flavonoids (Sak 2014). This type of activity has been observed in studies of vitexin, which inhibits apoptosis in noncancerous cells and acts as antioxidant while it has different effect on apoptosis in tumour cells (Babaei et al. 2020). For example, vitexin induced ROS production in a dose-dependent manner and increased the expression of autophagy marker proteins in colorectal cancer cells (Bhardwaj et al. 2017). In the study of active constituents of *Prosopis cineraria* (L.) Druce (Fabaceae), vitexin had dose- and time-dependent anti-proliferative activity in chronic myeloid leukaemia (K-562) cell line and induced apoptosis through reducing superoxide dismutase (SOD) activity and elevating ROS, nitric oxide (NO), and malondialdehyde (MDA) levels (Sarkar et al. 2020).

The prooxidant properties of flavonoids seem to be dependent on its concentration, cell type and culture conditions as well as on their chemical structure (Xiao et al. 2016). Generally, this type of activity relates to the compounds having multiple hydroxyl groups in ring B, but also there is evidence that the 2,3-double bond and 4-oxo arrangement of flavones may promote the formation of ROS induced by divalent copper in the presence of oxygen (Procházkova et al. 2011). However, even the minor modifications in the molecules can be responsible for strong variations in their activity, and flavonoids with very similar structures could not produce identical biological responses (Sak 2014).

The literature data describe the anticancer and proapoptotic activity of flavone C-glycosides towards different cell lines (vitexin – human leukaemia U937; vitexin and orientin – oesophageal cancer EC109; orientin – human cervical adenocarcinoma HeLa; isoorientin – liver hepatocellular carcinoma HepG2; vicenin-2 and orientin – prostate cancer cell lines PC-3, DU-145 and LNCaP) (Nagaprashantha et al. 2011; Lee et al. 2012; Zhu et al. 2012; Yuan et al. 2013, 2014; Guo et al. 2014). In the above-mentioned studies on proapoptotic activity of flavone C-glycosides, the IC_50_ values were higher than that required by the NCI. As a result of our research, all analysed flavone C-glycosides (vitexin, orientin, isoorientin, vicenin-1, vicenin-2 and vicenin-3) were considered inactive against the tested cell lines. However, the significant growth inhibitory activity of fraction C may be due to the presence of a whole flavone complex in high concentration. In the composition of fraction C, flavone monoglycosides, derivatives of apigenin and luteolin, constituted 19.5%, while their diglycosides 77.2%. Among the analyzed extracts the highest amount of flavone C-glycosides next to saponins per 1 mg of lyophilisate was quantified in fraction C. Aqueous-alcohol extract A (70% methanol) contained significantly lower amount of C-glycosylflavones, but only slightly less saponins. In contrast, the aqueous extract (B) with the weakest cytotoxic activity was distinguished by the lowest content of the secondary metabolites tested. It contained over 22 times less C-glycosylflavones compared to the fraction C and over 3 times less compared to the extract obtained using 70% methanol (A). The content of steroidal saponins in aqueous extract B was more than twice lower compared to extract A and fraction C. Taking into account the differences in chemical composition and the results of cytotoxic activity determinations, it can be concluded that the observed cytotoxic effects may be related to the synergy of action of flavones and saponins.

In the next stage of our work the role of selected cellular factors in induction of cancer cell death by the most active fraction C from fenugreek seeds was investigated. Studies included determining the rate of apoptosis, caspase-3/7 activity, modulation of mitochondrial membrane potential (MMP) and the production of reactive oxygen species (ROS).

So far, it has been proved that the mechanism of apoptosis induced by fenugreek seed extracts in Jurkat T-cell lymphoma cells (Al-Daghri et al. 2012), Hep2 liver cancer (Khalil et al. 2015) and MCF-7 breast (Khoja et al. 2011; Alshatwi et al. 2013) was based on caspases-3 and −8 (Al-Daghri et al. 2012; Alshatwi et al. 2013; Khalil et al. 2015), and caspase-9 (Khoja et al. 2011). It was found that apoptosis of MCF-7 breast cancer cells triggered by methanol extract from *T. foenum-graecum* seeds occurs as a result of stimulation of the external – receptor pathway (Alshatwi et al. 2013).

Our research indicated stronger proapoptotic activity of fraction C on SKOV-3 than HeLa cell line. Fraction C increases the activity of caspase-3/7 in both cell lines. Intracellular cysteine proteases – caspases play a key role in the mechanism of apoptosis, among which initiating caspases (2, 8, 9, 10) and executive caspases (3, 6, 7) can be distinguished (Korzeniewska-Dyl 2007, 2008). They are present in cells in the form of inactive zymogens – procaspase. Procaspases are activated by the induction of so-called external or/and internal apoptosis pathway. The external pathway of apoptosis induction (so-called receptor pathway) is associated with stimulation of the death receptor on the cell membrane and activation of the cascade of initiating and executive caspases, with procaspases-3 and −7 being activated by caspases-8 and −9 (Hordyjewska and Pasternak 2005; Stepień et al. 2007; Korzeniewska-Dyl 2007, 2008). The internal pathway of apoptosis, associated with mitochondria and endoplasmic reticulum, is activated under the influence of an increase in the concentration of reactive oxygen species, changes in the oxidoreductive potential in the cell, an increase in the concentration of Ca^2+^ ions in the cytoplasm, electrolyte disturbances and DNA damage (Hordyjewska and Pasternak 2005; Grądzka 2006; Stepień et al. 2007).

The loss of mitochondrial inner transmembrane potential is an indicator of cellular health and is often observed in the early stages of apoptosis. As a result of activation of the internal apoptosis pathway, the mitochondrial membrane potential decreases and cytochrome C leaks into the cytoplasm (Grądzka 2006; Łabędzka et al. 2006; Stepień et al. 2007). In our study, it was showed that fraction C modulates mitochondrial membrane potential (MMP) in SKOV-3 cells and this confirms the role of mitochondria in death process of these cells. No changes in MMP were observed in HeLa cells, although this does not deny that the mitochondrial pathway may be involved in the process of apoptosis.

Reactive oxygen species (ROS) play a significant role in cell regulation, activation of a signalling cascade and cell apoptosis. In our experiments, fraction C increases the production of ROS in HeLa and SKOV-3 cells. These results confirmed that this kind of cells death is induced by oxidative stress.

The conducted experiments allowed for the evaluation of some factors that play a role in the proapoptotic effect of the biologically active fraction C on HeLa and SKOV-3 cell lines. It was revealed that apoptotic populations occurred in the fraction treated cells and caspases-3 and −7 were involved in the cell death. Apoptosis may occur as a result of stimulation of the so-called internal pathway of apoptosis, in which changes in the mitochondrial potential of cells (SKOV-3 cell line) and increase in free radicals (oxidative stress) were observed.

In conclusion, the obtained results complement the current data on the cytotoxic activity of *Foenugraeci Semen* and its selected active compounds. For the first time, the proapoptotic effect of fenugreek seeds and the studied steroid sapogenins on SKOV-3 and MOLT-4 cell lines was revealed. So far, yamogenin has not been tested on cancer cells, and data on the cytotoxic effect of tigogenin have also been supplemented. Strong synergistic cytotoxic effect of flavonoids and the saponin complex contained in *T. foenum-graecum* was demonstrated. Further research is needed on the mechanism of action of this plant material and the extracts obtained from it in cells.

## Data Availability

The datasets are available from the corresponding author upon reasonable request.
